# Biocompatible nickel-prussian blue@silver nanocomposites show potent antibacterial activities

**DOI:** 10.4155/fsoa-2017-0048

**Published:** 2017-09-06

**Authors:** Sudip Mukherjee, Sourav Das, Saketh Nuthi, Chitta Ranjan Patra

**Affiliations:** 1 Chemical Biology Division, CSIR-Indian Institute of Chemical Technology, Uppal Road, Tarnaka, Hyderabad 500007, Telangana State, India; 2 Academy of Scientific & Innovative Research (AcSIR), Training & Development Complex, CSIR Campus, CSIR Road, Taramani, Chennai 600113, India

**Keywords:** antibacterial activity, biocompatible, CEA assay, nanocomposite, nickel-prussian blue nanoparticles, silver nanoparticles

## Abstract

**Aim:**

Silver nanoparticles have long been used as potent antibacterial agents. However, toxicity concerns of silver nanoparticles have limited their successful clinical applications. Hence, development of silver-based novel biocompatible nanomaterials for antibacterial applications is a challenging task.

**Materials & methods:**

Accordingly, in this work, we synthesized a biocompatible silver-based nanocomposite for antibacterial applications. The nanocompostie was characterized by several analytical techniques. The nanocomposite was further tested for its cytotoxicity in cells, chicken embryo and bacteria.

**Results & Conclusion:**

Herein, we report a simple and cost-effective method for the synthesis of nickel-prussian blue@silver nanocomposites. The nanocomposite is highly stable and shows biocompatibility observed by *in vitro* assay and by *ex vivo* chicken embryonic angiogenesis assay. The nanocomposite exhibits profound antibacterial activity toward Gram-negative (*Escherichia coli*) and Gram-positive (*Bacillus subtillus*) bacteria. The results altogether suggest the future potential applications of nickel-prussian blue@silver nanocomposite as an antibacterial agent.

## Lay abstract

Silver nanoparticles, being a potent antibacterial agent, hold enormous potential in the treatment of bacterial infections. However, serious toxicity concerns have limited their clinical use. Hence, design and development of biocompatible and alternative silver-based nanomedicine is urgently required. In this manuscript, we developed a biocompatible nickel-prussian blue@silver nanocomposite that demonstrates excellent antibacterial activity in both Gram-positive and Gram-negative bacteria and could be used as potent antibacterial agent in the near future.

Prussian blue nanoparticles (PBNPs) and their analogs have been used for various biomedical applications including drug delivery [1–3], imaging [4], MRI contrast agent [5], biosensors [6], photoacoustic imaging contrast agents [7] and photothermal ablation agents [8] due to their high biocompatibility, low production cost, ease of surface modification and high drug loading. Moreover, prussian blue complex (radiogardase, prussian blue in soluble form) is a US FDA-approved drug for the removal of radioactive and nonradioactive heavy metals (thallium and cesium) from the human body [9]. Low toxicity, long-term stability and low production cost would make prussian blue and its analogs ideal candidates for the treatment of various diseases [9].

Recently, bimetallic nanocomposites have gained enormous attention due to their multifunctional applications in healthcare and medicine. Presence of dual nanoparticles in nanocomposites can be useful to demonstrate multifunctional properties without hampering their own physical characteristics and applications. Very recently, scientists have synthesized core/shell nanocomposites of gold nanoparticles and prussian blue and its analog nanoparticles (Au@PB) and successfully used them for photoacoustic/CT bimodal imaging and photothermal ablation of cancer [10,11]. On the other hand, silver nanoparticles (AgNPs) are well established for their excellent antibacterial property [12,13]. AgNPs also have several applications (wound healing, water purification, medical application, wood preservatives, solar panels, etc.) in and out of biomedical sciences [14,15]. Several groups have previously reported the diverse biomedical applications (antibacterial activity, tissue engineering, antibioflim efficacy, photothermal therapy, photodynamic therapy, etc.) of different silver-doped nanocomposites including Ag-doped TiO_2_, hydroxyapatite silver nanocomposite, chitosan silver nanocomposites and so on [16–19]. With this in mind, combining the activities of AgNPs along with the prussian blue analogs could open a new dimension for successful fabrication of new multifunctional therapeutic agents. Surprisingly, there is no report of AgNP-based nanocomposites comprising prussian blue and its analogs, to the best of our knowledge.

Interestingly, various groups have reported that MRI could be utilized for *in vivo* tracking of bacteria as well as for characterizations of microbial infections inside human body [20,21]. Furthermore, MRI could be utilized for the imaging of tumors colonized with bacterial ferritin-expressing *Escherichia coli* [22]. Hence, the importance of MRI in detecting and characterizing bacterial infections is well established. In this context, we designed and developed a simple, ecofriendly and cost-effective method for the synthesis of nickel-prussian blue@silver nanocomposite (NiPB@AgNC). Selection of nickel-prussian blue (NiPB) is mainly due to the presence of its magnetic properties, which can be utilized for MRI applications [23]. Similarly, AgNPs were selected for their excellent antibacterial activity and long history of medicinal use since ancient times. Hence, the as proposed NiPB@AgNC should be useful for simultaneous detection and treatment of antimicrobial infections inside the body as a multifunctional agent. The nanocomposite was characterized by several physicochemical methods. We found that the nanocomposite is highly stable and biocompatible in nature. More interestingly, NiPB@AgNC exhibits excellent antibacterial activity toward Gram-negative (*E. coli*) and Gram-positive (*Bacillus subtillus*) bacteria. The results altogether suggest the future potential applications of NiPB@AgNC as an excellent antibacterial agent as well as a possible diagnostics agent for microbial infections.

## Experimental procedures

### Materials

Silver nitrate (AgNO_3_), sodium borohydride (NaBH_4_), potassium ferricyanide K_3_[Fe(CN)_6_)], nickel acetate [Ni(OAc)_2_], Dulbecco's Modified Eagle Medium (DMEM), phosphate-buffered saline (PBS), penicillin, streptomycin, kanamycin, fetal bovine serum, 3-(4,5-dimethylthiazol-2-yl)-2,5-diphenyltetrazolium bromide (MTT) were purchased from Sigma-Aldrich Chemicals, MO, USA. All the chemicals were used without further purification.

#### *Stock solution preparation*

10^-2^ (M) of AgNO_3_ and 10^-3^ (M) of K_3_[Fe(CN)_6_)] and [Ni(OAc)_2_] stock solution was prepared in sterile Millipore water and used for the synthesis of NiPB@AgNC.

#### Cell lines

Human lung cancer cell lines (A549) and mouse melanoma cell lines (B16F10) were purchased from American Type Culture Collection (ATCC). Human endothelial somatic hybrid cell lines (EA.hy926) were a kind gift from S Oglesbee, Tissue Culture Facility, University of North Carolina, Lineberger Comprehensive Cancer Center, NC, USA, and S Chatterjee, Anna University - K B Chandrasekhar, Chennai, India.

### Synthesis of AgNPs

AgNPs were synthesized by borohydride reduction methods according to the published literature [12]. Briefly, 25 ml of AgNPs was prepared by the reduction of 1 ml of AgNO_3_ (10^-2^ M) in 10 ml of water using 14 ml of NaBH_4_ (0.05 mg/ml) for 1 h under continuous stirring condition.

### Synthesis of NiPB@AgNC

In the first step of the reaction, 15 ml of 10^-3^ (M) of K_3_[Fe(CN)_6_)] and 15 ml of 10^-3^ (M) of [Ni(OAc)_2_] (1:1, v/v) were added slowly into 25 ml of AgNPs, under vigorous stirring for 1 h. The mixture of K_3_[Fe(CN)_6_)] and [Ni(OAc)_2_] (1:1, v/v) was repeatedly added to the resultant solution and allowed to stir for another 4 h in the following step to prepare yellow-colored NiPB@AgNCs.

### Stability studies of NiPB@AgNC in various physiological buffers & salines

The stability of any nanomaterials is very important before its use in healthcare. *In vitro* stability of NiPB@AgNC was investigated in 10% NaCl solution and different PBS with different pH (6, 7.4 and 8) for 2 weeks. Briefly, 100 μl of NiPB@AgNC pellet was incubated with 900 μl of respective buffers and kept for 14 days. The absorbance and λ_max_ of the corresponding mixtures was recorded in UV–Visible spectroscopy in a time-dependent manner. Finally, we plotted the λ_max_ of NiPB@AgNC (wavelength corresponds to maximum absorbance) incubated in various buffers in different time points and compared the stability of the nanocomposites. Additionally, we measured the hydrodynamic diameter of NiPB@AgNC incubated in various buffers using dynamic light scattering (DLS: Zetasizer Ver. 6.20; Malvern-MAL100442) in different time points to check the stability of the nanocomposites.

### Cell culture experiments

All cancer cell lines [A549 and B16F10] and normal cell line (EA.hy926) were cultured in DMEM media supplemented with 10% fetal bovine serum, 0.001% antibiotics (penicillin–streptomycin–kanamycin) and 5% L-glutamine in a humidified 5% CO_2_ incubator at 37°C for all *in vitro* biological experiments. The cells were incubated with different concentrations of NiPB@AgNC after 30 min of exposure under UV light.

### Cell viability test using MTT reagent

Initially, 10 × 10^3^ cells (EA.hy926, A549 and B16F10) were seeded in a 96-well plate and kept for 24 h in cell culture conditions (humidified 5% CO_2_ incubator at 37°C). Cell viability of all cancer (A549 and B16F10) and normal cells (EA.hy926) were carried out using MTT reagents after incubation of cells with different doses of NiPB@AgNC (1, 2.5, 5 and 7.5 μl/ml correspond to [Ag] = 14, 35, 70 and 105 μM, respectively) for 24 h according to our published protocol. After 24-h incubation, all treated cells were washed with PBS to remove surface-bound nanoparticles, and subsequently 100 μl of MTT solution (0.5 mg/ml in DMEM media) was added into each well for further 4 h. After 4 h of incubation, the MTT solution was replaced by freshly prepared 100 μl of DMSO:MeOH (1:1, v/v) in each well and absorbance were measured at λ_max_ = 575 nm. Finally, the results were expressed as normalized percent cell viability = ([A570 {treated cells} - background]/[A570 {untreated cells} - background]) × 100.

### Confocal microscopic analysis for fluorescent cytoskeleton & nucleus observation

To assess the morphology of the cells by polymerized actin, following fixation cells were analyzed by confocal microscopy. First, cells were grown on a 60-mm dishes on embedded cover slips at a density of approximately 50,000 NIH-3T3 cells/well (obtained from NCCS, Pune, India). After 18–24 h, the cells were treated with NiPB@AgNC (dose: 7.5 μl/ml; 105 μM with respect to silver). Following incubation for 24 h with the nanocomposite, the actin cytoskeleton was labeled according to the manufacturer's instructions. Briefly, cells were washed with PBS, fixed with 3.7% formaldehyde solution for 10 min, washed with PBS and permeabilized by 0.1% Triton X-100 in PBS. The cells were then washed with PBS and blocked with 1% BSA for 30 min (to avoid nonspecific binding). After washing with PBS, the cells were incubated for 20 min with 5 μl of Alexa Fluor 488 phalloidin (Thermo Fisher Scientific, MA, USA, Cat no. A12379) from a 200 units/ml methanolic stock. Cells were finally washed, fixed and mounted on a DAPI (4′,6-diamidino-2-phenylindole) vectashield and observed under confocal imaging [laser used, 404.2, 488 nm in Nicon Eclipse Microscope].

### Chick embryonic angiogenesis assay

The chicken embryonic angiogenesis (CEA) assay, a standard *ex vivo* angiogenesis assay, has been widely used to study angiogenesis and tumor invasion of different cancers [24,25]. The fertilized chicken eggs were purchased from government poultry (Directorate of Poultry Research, Hyderabad, Telangana) and incubated in an egg incubator (Southern) at 37°C and 60–70% relative humidity. On the fourth day of incubation, a small gap was created on the shell using forceps carefully, sterilized filter-paper discs were soaked in the NiPB@AgNC pellet ([Ag] = 3.5, 7, 14 μM). The discs were employed around the blood vessels after exposing the gap area that was covered with parafilm later. The treated and untreated eggs were incubated for next 4 h in the incubator. The images were captured at 0 and 4 h post-treatments using an LEICA camera (LEICA MC120-HD) attached to a LEICA stereomicroscope (LEICA S8AP0) at 10 megapixel magnifications.

### Antibacterial activity of NiPB@AgNC

Gram-negative *E. coli* and Gram-positive *B. subtillus* growth was maintained at 37°C in shaker in Luria bertani broth media. The inhibition growth of *E. coli* and *B. subtillus* treated with different doses of NiPB@AgNC (1, 2.5 and 5 μl/ml corresponding to [Ag] = 14, 35 and 70 μM; respectively) was monitored by multimode spectrophotometer (Varioskan) at 600 nm up to 6 h. Streptomycin (100 μg/ml) was used as positive control antibiotics. Agar disc diffusion technique was used to evaluate antibacterial activity of *E. coli* and *B. subtillus* against nanocomposite and standard antibiotics like streptomycin (100 μg/ml) and penicillin (100 μg/ml) by measuring the inhibitory zone.

### Release of silver ions from NiPB@AgNC in PBS

Release of silver ions from nanocomposite was determined using inductively coupled plasma optical emission spectrometry (ICP-OES) analysis. Initially, 500 μl of the ash yellow pellet of NiPB@AgNC was obtained after centrifugation of 50 ml of reaction mixture at 17,700 r.p.m. 15°C for 40 min. 100 μl of the pellet of the nanocomposite was incubated with 900 μl of PBS (pH 7.4) and kept for different time points (0–48 h). After each time point, the mixture was mixed with 20 ml of Milli-Q water and the solution was ultracentrifuged at 24,000 r.p.m. for 1 h at 20°C. The supernatant of each solution was collected and further submitted to ICP-OES analysis for the measurement of released silver content.

### Characterization techniques

The as-synthesized nanocomposite was thoroughly characterized by several physicochemical techniques. Initially, 50 ml of NiPB@AgNC (obtained after second step of reaction) was centrifuged for 40 min at 17,700 r.p.m. at 15°C using ultracentrifuge (Thermo Fisher Scientific, Sorvall-WX ultra 100). The intense loose ash yellow-colored pellet obtained after centrifugation was used for further characterizations and cell viability studies and antibacterial studies. The absorbance, crystallinity and purity of NiPB@AgNC were monitored by UV–Visible spectroscopy (JASCO dual-beam spectrophotometer [Model V-570]) and x-ray diffraction (XRD) analysis using Bruker AXS D8 Advance Powder x-ray diffractometer (CuKαλ = 1.5406 Å radiation), respectively. The size, morphology and surface charge (zeta potential) were determined by transmission electron microscopy (TEM: FEI Tecnai F12, Philips Electron Optics, Eindhoven, Holland) and DLS, respectively. ICP-OES [(iCAP-6500 DUO), Thermo Fisher Scientific, Cambridge, UK] was used for the determination of silver, nickel and iron in the loose pellet.

## Results & discussion

**Figure 1.**
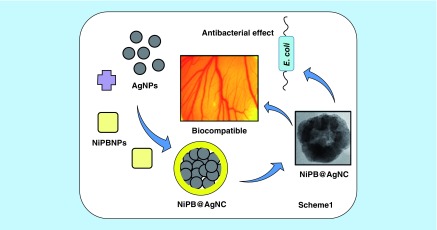
**Schematic representation of the synthesis, characterization and antibacterial application of nickel-prussian blue@silver nanocomposite.**

The synthesis of NiPB@AgNCs was carried out by simple addition of freshly prepared AgNPs with the mixture of equal volume of K_3_[Fe(CN)_6_)] [10^-3^(M)] and [Ni(OAc)_2_] [10^-3^(M)]. Initially, AgNPs were synthesized by borohydride reduction methods as the published literature [12]. AgNPs were prepared by the reduction of AgNO_3_ using NaBH_4_ as a reducing agent for 1 h under continuous stirring condition. In order to synthesize NiPB@AgNC, 15 ml of each 10^-3^ (M) of K_3_[Fe(CN)_6_)] and [Ni(OAc)_2_] (1:1, v/v) was added slowly into AgNPs (25 ml), under vigorous stirring for 1 h in the first step of the reaction. The mixture of K_3_[Fe(CN)_6_)] and [Ni(OAc)_2_] (1:1, v/v) was further added to the resultant solution for another 4 h to obtain intense yellow-colored NiPB@AgNCs. The addition of the mixture of equal volume of K_3_[Fe(CN)_6_)] and [Ni(OAc)_2_] generates *in situ* double-layer coating of NiPBNPs, which acts mostly as the outer framework of NiPB@AgNC. The NiPB@AgNC was characterized by several physicochemical techniques and found to be biocompatible in nature. Furthermore, NiPB@AgNC was utilized for antibacterial applications against Gram-negative (*E. coli*) and Gram-positive (*B. subtillus*) bacteria. The overall synthesis, characterization and antibacterial application for NiPB@AgNC is schematically represented in Figure 1.

### UV–visible spectroscopy

**Figure 2.**
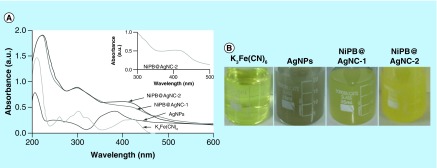

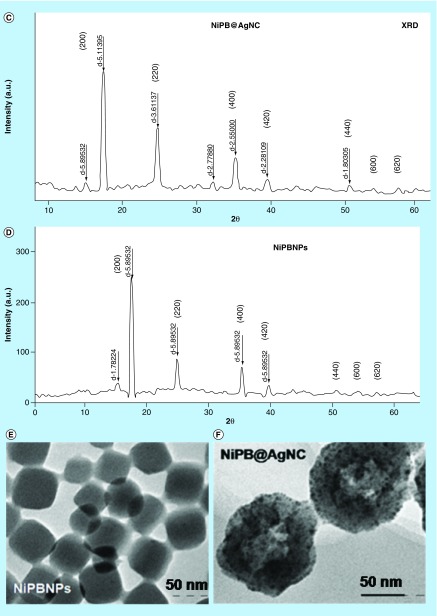

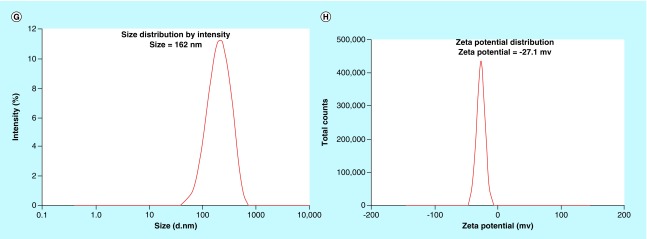
**Characterization techniques of nanocomposite.** **(A)** UV–visible spectrum of K_3_Fe(CN)_6_, AgNPs, NiPB@AgNC-1 and NiPB@AgNC-2; **(B)** optical color images of K_3_Fe(CN)_6_, AgNPs, NiPB@AgNC-1 and NiPB@AgNC-2; **(C & D)** x-ray diffraction pattern of NiPB@AgNC and NiPBNPs and **(E & F)** transmission electron microscopy images of NiPB@AgNC and NiPBNPs, **(G)** DLS size (hydrodynamic diameter in nm) and **(H)** zeta potential (in mV) of NiPB@AgNC. AgNP: Silver nanoparticle; DLS: Dynamic light scattering; NiPB@AgNC: Nickel-prussian blue@silver nanocomposite; NiPBNP: Nickel-prussian blue nanoparticle.

UV–visible spectroscopy was used to monitor the formation of NiPB@AgNC. Figure 2A shows the UV–visible spectrum of K_3_Fe(CN)_6_, naked AgNPs and NiPB@AgNCs obtained at first and second step of reaction (NiPB@AgNC-1 and NiPB@AgNC-2). The absorbance of NiPB@AgNC-1 and NiPB@AgNC-2 was monitored at λ_max_ approximately 410–415 nm indicating the possibility of formation of NiPB@AgNCs, which differs from the UV spectrum of K_3_Fe(CN)_6_ and AgNPs (Figure 2A). The absorbance intensity of NiPB@AgNC-2 is more compared with NiPB@AgNC-1, indicating the higher concentration of NiPBNPs after second step (Figure 2A). Figure 2B shows the optical images of the color of the K_3_[Fe(CN)_6_)] (light yellow), AgNPs (intense ash), NiPB@AgNC-1 (ash yellow) and NiPB@AgNC-2 (intense yellow), respectively, which emulate the plausible stepwise formation of nanocomposites.

### XRD spectroscopy

The crystal structure of NiPB@AgNC and NiPBNPs was identified by XRD analysis (Figure 2C & D). The diffraction peaks observed for both NiPB@AgNC and NiPBNPs at 200, 220, 400, 420, 440, 600 and 620 indicate face-centered cubic (Fm-3m) crystalline lattice structure [26,27] (Figure 2C & D). This result corroborates with the XRD pattern of well-established prussian blue-based nanoparticles and nanocomposites [26,27]. However, we did not find any separate diffraction peaks for AgNPs in the XRD spectra of NiPB@AgNC. This is probably due to the double-layer thick coating of NiPBNPs on AgNPs that mask the indicative peaks of AgNPs in the XRD spectra of NiPB@AgNC. This also might be due to the poor crystallinity of AgNPs [28]. XRD results further reveal that there is no change of crystal structure of basic moiety (NiPBNPs) after formation of the nanocomposites.

### Size, shape & morphology analysis

In order to find out the size and morphology of nanoparticles and nanocomposite (NiPB@AgNC), TEM analysis was carried out. Figure 2E shows the TEM images of NiPBNPs. TEM images of NiPBNPs show the cubic shape with size range approximately 50–75 nm, whereas AgNPs show mainly spherical shaped with size range of 20–40 nm (data not shown) [12]. On the other hand, the TEM images of NiPB@AgNC show nice spherical bimetallic nanocomposite structures with the size range of approximately 100–120 nm, which are ideal for the biomedical applications (Figure 2F). The DLS method was employed to calculate the hydrodynamic radii of nanocomposite. Result shows that the hydrodynamic diameter of NiPB@AgNC is approximately 162 ± 5.2 nm (Figure 2G). Again, the surface charge or zeta potential (ξ) is a crucial parameter for the dispersion or colloidal stability of nanoparticles. The size of the nanocomposite obtained from DLS is slightly higher compared with size obtained from TEM. DLS studies give an idea about the hydrodynamic diameter of any nanoparticles, which includes the association of water other molecules, whereas TEM shows the exact size of nanoparticles excluding the surface-attached molecules. The ξ value of NiPB@AgNC is highly negative (-27.1 ± 4.34 mV), indicating exceptional stability due to high-repulsive surface negative charge (Figure 2H).

### Fourier transformed infrared spectroscopy

**Figure 3.**
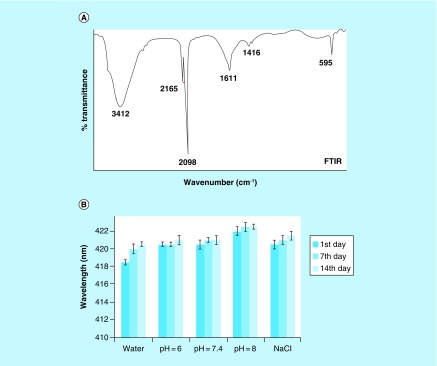
**FTIR and stability studies.** **(A)** FTIR spectra of nickel-prussian blue@silver nanocomposite and **(B)** stability studies of nickel-prussian blue@silver nanocomposite in different physiological buffers and solutions. FTIR: Fourier transformed infrared.

The peak at 2098 cm^-1^ in Fourier transformed infrared (FTIR) spectrum of NiPB@AgNC is indicating the presence of C≡N stretching frequency (Figure 3A) [29]. The lower stretching frequency of C≡N might occur due to the interaction of nickel with cyanide (C≡N) in the lattice state of NiPBNPs [3,29]. The cyanobridged complexes can easily be identified as they exhibit sharp peak at ν(CN), at 2000–2200 cm^-1^ [30]. The two peaks at 2098 and 2165 cm^-1^ arose owing to the transformation of the microstructure Fe^2+^–CN–Ni^3+^ to the structure Fe^3+^–CN–Ni^2+^. The peaks at 2098 and 2165 cm^-1^ correspond to the ν(Fe^2+^–CN–Ni^3+^) and ν(Fe^3+^–CN–Ni^2+^), respectively. More importantly, the major part of the complex contains trivalent ion rather than the bivalent ion. The FTIR peaks at 3412 and 1611 cm^-1^ designated to the O-H stretching and O-H bending vibrations of the crystal water in the framework of the nanocomposite, respectively [31,32]. The stretching frequency of the -COO bond comes at 1416 cm^-1^, which is less intense as nickel acetate is used as one of the precursor. Meanwhile, in the low-frequency region, the peak at 595 cm^-1^ corresponded to the Ni-O vibration in the NiO_6_ coordinate sphere [31,33].

### Inductively coupled plasma optical emission spectrometer

The concentration of metal ions (Ag: silver, Ni: nickel, Fe: iron and K: potassium) in NiPB@AgNC pellets was quantitatively estimated by ICP-OES analysis. The results show that the concentrations (in mg/l) of Ag, Ni, Fe and K in NiPB@AgNC are 0.30, 0.09, 0.22 and 0.07, respectively. We have selected 1, 2.5, 5 and 7.5 μl/ml doses for the *in vitro* cell viability assay and antibacterial studies. The concentrations of silver present in those corresponding doses calculated from ICP-OES analysis of NiPB@AgNC are 14, 35, 70 and 105 μM, respectively.

### Stability studies

The stability of nanoparticles/nanocomposites is vital before its use in biomedical applications. Therefore, *in vitro* stability of nanocomposite was investigated in 10% NaCl solution and PBS with different pH (6, 7 and 8) for 2 weeks. Figure 3B shows that the maximum wavelength (λ_max_) of NiPB@AgNC in different buffers remains almost unchanged (<5 nm) after 2 weeks of incubation with respect to 24-h sample, which reflects the high colloidal stability. Here, it is important to mention that salt stability is an important parameter to determine the stability and to test the aggregation properties of nanoparticles. Several groups including ours have tested the *in vitro* stability of AgNPs/nanocomposites in salt or saline solutions [12]. However, it is vital to mention that bare AgNPs may show formation of AgCl in saline solutions with time and subsequent decrease in the absorbance (sign of aggregation) of the nanoparticles that indicate instability. In our case, we do not found any decrease in the absorbance of the nanocomposites incubated with saline solutions till 14 days. This indicates the stability of NiPB@AgNC in salt solutions. This is probably due to the external double-layer thick coating of NiPBs on AgNPs, which prevents the quick dissolution of AgNPs and subsequent formation of AgCl in salt solutions.

Moreover, we measured the hydrodynamic size of NiPB@AgNC in these buffers till day 14 (Table 1). From the Table 1, it is clear that no significant change in the hydrodynamic size of NiPB@AgNC incubated in different buffers has occurred till day 14. This directly supports the *in vitro* stability of NiPB@AgNC in various buffers (with pH 6–8) and saline solutions.

**Table 1.** **Hydrodynamic diameter of nickel-prussian blue@silver nanocomposite in different buffers in a time-dependent manner to check the stability.**

**Buffer solution**	**HD (0 days) in nm**	**HD (7 days) in nm**	**HD (14 days) in nm**
Water	162 ± 5.2	163 ± 6.9	167 ± 7.1

pH 6	171 ± 7.1	171 ± 1.2	175 ± 3.5

pH 7.4	165 ± 2.6	168 ± 3.9	168 ± 5.4

pH 8	170 ± 5.4	170 ± 2.9	171 ± 3.2

NaCl	171 ± 3.9	172 ± 4.1	174 ± 1.8

HD: Hydrodynamic diameter.

### *In vitro* cytotoxicity & morphological analysis

**Figure 4.**
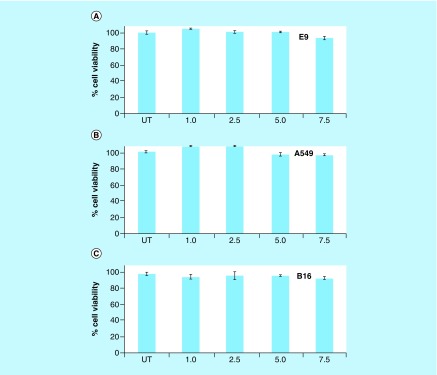
***In vitro* cytotoxicity of nanocomposite.** Cell viability assay of nickel-prussian blue@silver nanocomposite in **(A)** EA.hy926 (E9), **(B)** A549 and **(C)** B16F10 with different doses in for 24 h using 3-(4,5-Dimethylthiazol-2-yl)-2,5-diphenyltetrazolium bromide reagent. Numerical values indicate the volume of nickel-prussian blue@silver nanocomposite in μl/ml (where 1, 2.5, 5 and 7.5 μl/ml correspond to [Ag] = 14, 35, 70 and 105 μM, respectively).

For successful application of NiPB@AgNC in biology and medicine, it is very important to check their potential cytotoxicity. *In vitro* cell viability assay using MTT reagents is a basic assay to evaluate the cytotoxicity of any nanomaterials or drugs. In order to check the cytotoxicity of nanocomposites, we carried out *in vitro* cell viability assay using MTT reagents in various cancer (A549 and B16F10) and normal cell lines (EA.hy926) for 24 h in a dose-dependent manner (Figure 4A–C). Cell viability results show that NiPB@AgNC is biocompatible in nature up to high doses of silver ([Ag] = 105 μM) in both normal as well as cancer cells (Figure 4A–C). The biocompatibility of the synthesized nanocomposite could make it useful for further biomedical applications. Our previous report demonstrates that AgNPs show cytotoxicity at 30 μM dose in various normal cell lines [12]. In this case, the nanocomposite is biocompatible even at higher concentration of silver ([Ag] = 105 μM), indicating the biocompatibility of this material up to high doses.

**Figure 5.**
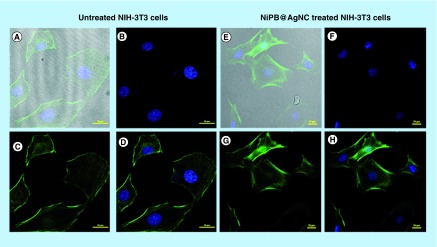
**Confocal cell images of NIH-3T3 cells stained with DAPI and Alexa Fluor 488 phalloidin.** **(A–D)** NIH-3T3 cells were kept as untreated. **(A)** Merged images of all filters, **(B)** DAPI stained for nucleus of untreated cell, **(C)** Alexa Fluor 488 phalloidin staining for actin filaments and **(D)** merged images of blue and green filter of untreated cells. **(E–H)** Confocal images of NIH-3T3 cells were incubated with NiPB@AgNC (7.5 μl/ml corresponds to [Ag] = 105 μM) for 24 h). **(E)** Merged images of all filters, **(F)** DAPI stained for nucleus, **(G)** Alexa Fluor 488 phalloidin staining for actin filaments and **(H)** merged images of blue and green filter. NiPB@AgNC: Nickel-prussian blue@silver nanocomposite.

To provide further evidence of biocompatibility and visualization of cell morphology upon treatment with nanocomposites, confocal microscopy was used. The cell morphology and observation of fluorescent cytoskeleton and nucleus also give the idea of the biocompatibility of the nanoparticles or nanocomposite-treated cells. Staining of the actin filaments and nucleus give the overall structure of the cells. Results reveal that there is no change in the overall morphology of the NIH-3T3 cells even in the treatment of high doses of NiPB@AgNC (7.5 μl/ml; correspond to [Ag] = 105 μM for 24 h) compared with the untreated cells (Figure 5A–H). The pictures clearly show that there is no change in the actin cytoskeleton structure or presence of nuclear fragmentation even after high doses of treatment (Figure 5A–H). These results indicate the biocompatibility of nanocomposites even at high doses [34,35].

### CEA assay: *ex vivo* toxicity

**Figure 6.**
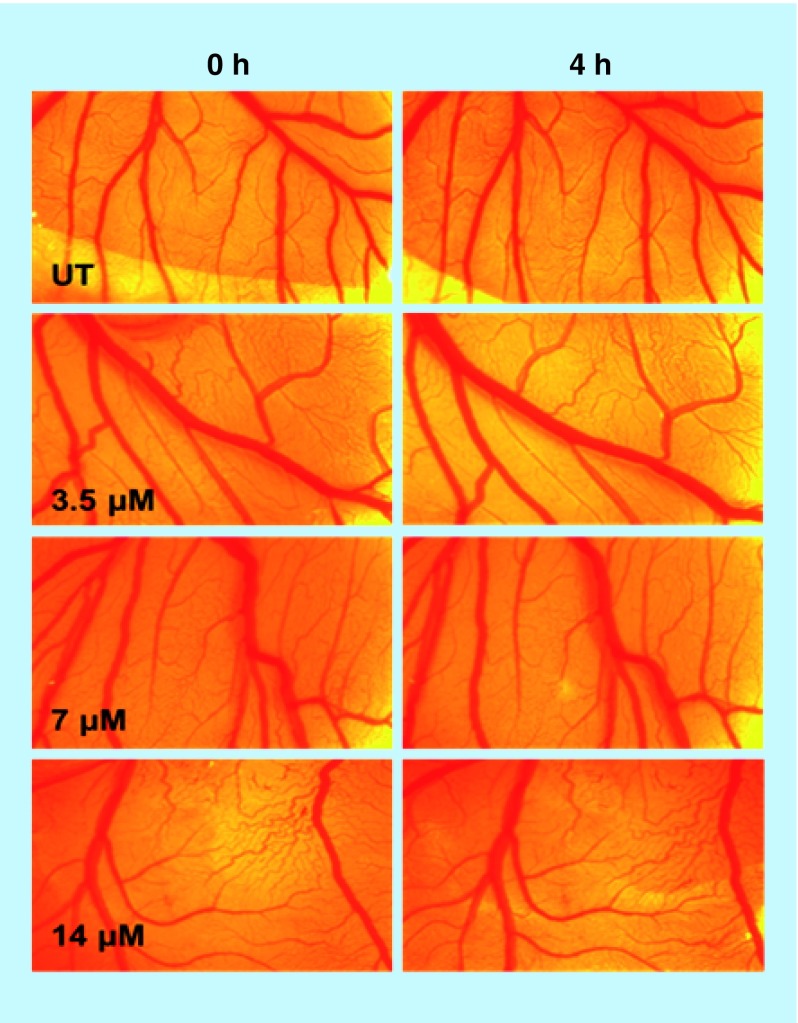
***Ex vivo* chicken embryonic angiogenesis assay in presence of nickel-prussian blue@silver nanocomposite at different doses (with respect to silver concentration) in a time-dependent manner.**

The CEA assay, a typical *ex vivo* angiogenesis assay, has been commonly used to study angiogenesis and *ex vivo* toxicity [24,25]. In order to investigate the *ex vivo* biocompatibility of the nanocomposite we performed the CEA assay. Figure 6 indicates the CEA assay where egg yolk was untreated control (control: Figure 6), or treated with NiPB@AgNC at different concentrations (3.5, 7 and 14 μM). The incubation was done for 4 h. From the CEA data, no remarkable change in the blood vessels is found in different doses. The CEA results further support the *ex vivo* biocompatible nature of the nanocomposite, which could be useful for the safe use of these nanomaterials in healthcare and medicine after proper preclinical study. Hence, both *in vitro* and *ex vivo* results support the nontoxic nature of NiPB@AgNC.

### Antibacterial activity

#### Growth inhibition study

**Figure 7.**
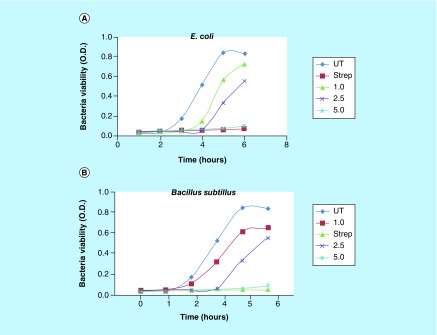
**Antibacterial activity of nanocomposite.** Liquid growth inhibition kinetics of **(A)** *Escherichia coli* and **(B)** *Bacillus subtillus* using different volumes of nickel-prussian blue@silver nanocomposite. The numerical value indicates the volume of nickel-prussian blue@silver nanocomposite in μl/ml (where 1, 2.5, 5 μl/ml correspond to [Ag] = 14, 35 and 70 μM, respectively).

Time-dependent growth of untreated bacteria (*E. coli* and *B. Subtillus*) and bacteria treated with different doses of NiPB@AgNC in Luria bertani broth was studied by measuring the O.D. of bacteria. Figure 7A & B shows the graphical representation of inhibition kinetics for *E. coli* and *B. subtillus* treated with different concentrations of nanocomposite. The inhibition of growth is observed with NiPB@AgNC in a dose-dependent manner compared with untreated bacteria. Around 100% inhibition is observed at 70 μM of Ag (5 μl/ml of NiPB@AgNC) at 6 h, which is similar with the effects of positive-control drug streptomycin (100 μg/ml), indicating high therapeutic efficacy.

#### Zone of inhibition study

**Figure 8.**
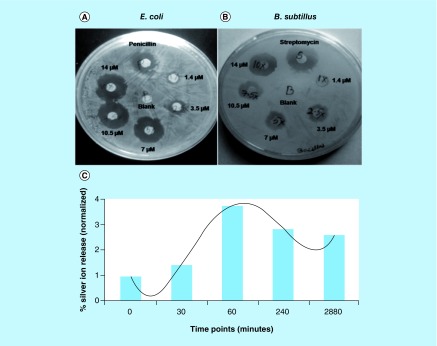
**Zone of inhibition and silver ion release study.** Zone of inhibition of nickel-prussian blue@silver nanocomposite with different doses in **(A)** *Escherichia coli* and **(B)** *Bacillus subtillus*. Streptomycin and penicillin were kept as positive control. **(C)** Silver ion release kinetic study in phosphate buffer (pH 7.4) using inductively coupled plasma optical emission spectrometry analysis.

Agar diffusion disc method was employed in order to investigate the antibacterial activity of different concentration of NiPB@AgNC toward *E. coli* and *B. subtillus*. The antibacterial activity of NiPB@AgNC was compared with standard antibiotics like streptomycin (100μg/ml) and penicillin (100 μg/ml) by measuring the zone of inhibition (Table 2, Figure 8A & B). The results are tabulated in Table 2. Interestingly, in case of *E. coli*, NiPB@AgNC (at [Ag] = 14 μM) shows larger inhibition zone (∼20 mm) compared with positive-control drug penicillin (∼14 mm; Figure 8A & B). Again, NiPB@AgNC shows almost comparable inhibition zone (∼16 mm) with standard antibiotics streptomycin (∼18 mm) in Gram-positive bacteria, *B. subtillus* (Figure 8A & B). Our earlier published reports show that AgNPs exhibit around 10 mm of inhibition zone toward *E. coli* at 30 μM of silver concentration [12]. Compared with earlier published reports, it is obvious that the nanocomposites exhibit twice the antibacterial activity (∼20 mm zone of inhibition at [Ag] = 14 μM), at half of the silver concentration compared with AgNPs. The nanocomposites in the present work thus show far superior antibacterial activity compared with AgNPs, establishing the elevated therapeutic efficacy of these materials.

**Table 2.** **Zone of inhibition values of nickel-prussian blue@silver nanocomposite toward *Escherichia coli* and *Bacillus subtillus*.**

**Dose of NiPB@AgNC in μM with respect to Ag**	**ZOI toward *E. coli* (in mm)**	**ZOI toward *B. subtillus* (in mm)**
14	20	16

10.5	19	13

7	18	11

3.5	13	11

1.4	7	7

NiPB@AgNC: Nickel-prussian blue@silver nanocomposite; ZOI: Zone of inhibition.

It is well established that AgNPs show excellent antibacterial activity due to mainly bacterial membrane damage or production of reactive oxygen species by Ag^+^ ions [12]. To get a possible idea regarding the mechanism of antibacterial activity of the nanocomposites, we checked the release of silver ions in a time-dependent manner. Results show that a time-dependent release of Ag^+^ ions from NiPB@AgNC, which get saturated after 60 min and continue to sustain release till 48 h (Figure 8C). This sustained release of Ag^+^ ions could be the plausible reason for the antibacterial activity of the nanocomposites in Gram-negative and Gram-positive bacteria. Recently, Lu *et al*. exhibited that AgNPs convert to Ag^+^ and superoxide anions in presence of dissolved oxygen in a rapid manner, according to the following reaction [36,37]:

AgNPs + O_2_ (dissolved oxygen) → Ag^+^ (silver ion) + O_2_^.-^ (superoxide radical)

However, the release of Ag^+^ ions from AgNPs is rapid and hence nonspecific in nature. Hence, rapid release of Ag^+^ may cause toxicity due to lack of targeting ability, nonspecific deposition of silver and lack of stability. However, the release of Ag^+^ from NiPB@AgNC is much slower than AgNPs, due to double-layer coating of NiPBNPs on AgNPs. Consequently, this prevents rapid and nonspecific leakage of Ag^+^ ions. Hence, this sustained and slow release of Ag^+^ ions from NiPB@AgNC ensures the enhanced therapeutic efficacy (antibacterial activity) and less cytotoxicity (in normal cells). Recently, different groups including ours have demonstrated development of multifunctional nanoparticles/nanocomposites for various theranostics applications [38–40]. Together, these above results strongly support the successful fabrication of a robust nanocomposite that can be used for bacterial theranostics applications.

## Conclusion

In this article, we report the synthesis of NiPB@AgNCs. The synthesis method is simple, efficient, ecofriendly and economically cheap. Physicochemical characterizations of nanocomposite show high stability. The *in vitro* MTT assay in normal and cancer cells demonstrate the biocompatibility nature of nanocomposite. *Ex vivo* CEA assay further supports the biocompatibility of the nanomaterials. Finally, the nanocomposite exhibits excellent antibacterial activity against Gram-negative (*E. coli*) and Gram-positive (*B. subtillus*) bacteria. The results altogether demonstrate the future potential applications of this newly developed nanomedicine (NiPB@AgNC) as an effective antibacterial agent.

## Future perspective

These exciting data on potent antibacterial activity of highly biocompatible silver-based nanocomposites are likely to pave the way for the development of potential safer nanomedicine that can be effectively used for clinical studies. Our synthesized nanomedicine (NiPB@AgNC) is safer at higher doses compared with several commercial silver-based nanomedicines. Moreover, NiPB@AgNC demonstrates significant high therapeutic activity. This could be a game changer in the near future considering low toxicity and improved therapeutic efficacy. However, detailed and rigorous studies still need to be explored, mainly regarding its mechanistic aspects and systematic *in vivo* toxicity before its clinical applications. Additionally, MRI studies also need to be performed to confirm the potential diagnostic ability of NiPB@AgNC for bacterial infections.

**Summary points**

- Nickel-prussian blue@silver nanocomposite (NiPB@AgNC) has been synthesized by a simple, efficient and cost-effective method.
- NiPB@AgNC has been characterized by several analytical techniques (UV–visible spectroscopy, x-ray diffraction, dynamic light scattering and transmission electron microscopy, etc.).
- The nanocomposite is highly stable in various physiological buffers and solutions for 2 weeks.
- NiPB@AgNC is biocompatible in nature, observed by *in vitro* (cellular morphological analysis, cytoskeleton analysis, nucleus analysis and 3-(4,5-dimethylthiazol-2-yl)-2,5-diphenyltetrazolium bromide assay) and *ex vivo* chicken embryonic angiogenesis assay.
- The nanocomposite exhibits potent therapeutic efficacy (antibacterial activity) against Gram-negative (*Escherichia coli*) and Gram-positive (*Bacillus subtillus*) bacteria.
- Mechanistic studies revealed that low cytotoxicity and improved antibacterial efficacy is due to the slow and sustained release of the silver ion (Ag^+^).
